# Phenotypic hip and elbow dysplasia trends in Rottweilers and Labrador retrievers in South Africa (2007–2015): Are we making progress?

**DOI:** 10.4102/jsava.v88i0.1534

**Published:** 2017-11-22

**Authors:** Robert M. Kirberger

**Affiliations:** 1 Department of Companion Animal Clinical Studies, University of Pretoria, South Africa

## Abstract

Canine hip and elbow dysplasia are major orthopaedic problems prevalent the world over, and South Africa is no exception. Hip and elbow dysplasia phenotypic status is certified by a number of different radiographic schemes in the world. South Africa uses the Fédération Cynologique Internationale system to certify hips, and the International Elbow Working Group scheme to certify elbows. One way of reducing these often crippling conditions is by selective breeding using only dogs with no or marginal dysplastic joints. In South Africa, only seven breeds, including the Rottweiler, have breeding restrictions for hip dysplasia. There are no such restrictions for elbow dysplasia. This study assessed the prevalence of hip and elbow dysplasia over a 9-year-period in the Rottweiler and the Labrador retriever in South Africa as evaluated by official national scrutineers. Records from 1148 Rottweilers and 909 Labrador retrievers were obtained and were graded as normal or dysplastic, and numerical values were also evaluated. Data were compared between the two breeds, males and females as well as over time and were compared with similar data of the Orthopaedic Foundation for Animals in the United States. The prevalence values for hip dysplasia in Rottweilers and Labrador retrievers were 22% and 31%, respectively, whereas for elbow dysplasia the values were 39% and 19%, respectively. In Labrador retrievers, this incidence was much higher than in the American population. Rottweiler hip and elbow dysplasia numerical scores significantly improved over time, whereas in Labrador retrievers, only hip dysplasia showed a minor but significant improvement. This study proved that prescribing minimum breeding requirements, as in the Rottweiler in this study, significantly improved the breeding stock, suggesting that minimum hip and elbow breeding requirements should be initiated for all breeds at risk of these often crippling conditions.

## Introduction

Canine hip dysplasia (HD) and elbow dysplasia (ED), the abnormal development of the hip and elbow joints followed by irreversible progressive arthrosis, are two of the most prevalent developmental orthopaedic conditions affecting dogs worldwide. According to data of the Orthopaedic Foundation for Animals (OFA) in the United States, 20.2% of Rottweilers and 11.5% of Labrador retrievers had HD from 1974 to 2015. The OFA values from 1974 to 2015 for dysplastic elbows were 36.7% for Rottweilers and 10.2% for Labrador retrievers. HD was first described in 1935 in a United States Kennel Gazette by Schnelle as reported in the late 1950s (Henricson & Olsen 1959) and over the years has crippled many dogs and has been the subject of numerous scientific reports and investigations with the search for better diagnostic and breeding selection information continuing unabated. The only way to markedly improve the welfare of HD and ED susceptible breeds is through phenotypic selection (Lewis, Blott & Woolliams 2013).

Hip dysplasia is characterised by the development of varying degrees of hip joint laxity with the subsequent development of coxarthrosis in most breeds. Clinical signs may start as young as 5 months of age (Woolliams, Lewis & Blott 2011). The prevalence of HD may be up to 70% in some breeds. It mainly affects larger breed dogs and is of particular concern in working dogs (Orthopaedic Foundation for Animals 2016).

Canine HD and ED have a polygenic mode of inheritance, although ED may also have a major gene involved, and its expression can be markedly influenced by the environment (Mäki et al. 2002, 2004; Worth, Bridges & Jones 2011). To date, radiological methods have been the mainstay of determining the phenotypic status of dogs to be used for breeding (Worth et al. 2011). A variety of radiographic HD screening programmes exist worldwide (Von Pückler, Tellhelm & Kirberger 2016; Worth et al. 2011). HD certification schemes typically use the standard ventrodorsal hip extended view as initially described in a report of the Hip Dysplasia Committee of the Fédération Cynologique Internationale (FCI) in Europe (Brass et al. 1978) as well as for submissions to the OFA in the United States (Rendano & Ryan 1985). In some countries, a ventrodorsal hip flexed view, also known as the ‘frog-leg’ view, may also be submitted and is a standard requirement for South Africa (Von Pückler et al. 2016). The degree of HD changes are given in up to 10 subcategories (FCI, OFA and others) or each hip is given a numerical score as per the British Veterinary Association (BVA)/Kennel Club scheme with a maximum score of 53 per hip (Flückiger 2008; Von Pückler et al. 2016). The latter scheme is also used by Australia and New Zealand. The FCI system generally accepts that Grades A and B are non-dysplastic dogs with some countries having a grade subdivision making B2 a marginally dysplastic dog (Malm et al. 2010). This is similar to the OFA system of excellent, good and fair for non-dysplastic dogs, borderline, and then dysplastic dogs graded as mild, moderate and severe (Keller, Dziuk & Bell 2011). Borderline cases are recommended by the OFA to have repeat radiographs taken after 6 months to either upgrade or downgrade them. The OFA and FCI systems can thus be equated using the following guidelines, where excellent equates to a FCI score of A1, good is A2, fair is B1, borderline is B2, mild is C, moderate is D and severe equates to E (Flückiger 2008; Von Pückler et al. 2016).

In South Africa, radiographic HD certification, initially graded as 0–4, has been done by the Faculty of Veterinary Science of the University of Pretoria since the mid-1960s. In the 1970s, a panel of four veterinarians certified HD radiographs on behalf of the South African Veterinary Association (SAVA). With the qualification of the first specialist veterinary radiologist in South Africa in 1977, the Kennel Union of South Africa (KUSA) started only accepting certificates issued by specialist veterinary radiologists. In January 2007, in order for the scheme to be internationally recognised, the FCI scheme was adopted for HD certification and the scheme is a combined one run by SAVA and KUSA. The grading is from A to E, with an additional subdivision for each grade of 1 or 2. The best hip is thus A1 and the worst hip E2. Currently, there are six specialist veterinary radiologists certifying dogs as individuals for the SAVA/KUSA/FCI scheme and an appeal process is in place as per the FCI guidelines. One other scheme is in place in South Africa. The German Shepherd Dog Federation has all its dogs graded by a single specialist radiologist according to the Verein für Deutsche Schäferhunde guidelines. To date, no articles exist that have looked at HD data over time for any dog breed in South Africa, and this publication will be limited to Rottweiler and Labrador retriever HD certifications evaluated according to the FCI scheme.

The polygenic nature of HD is because of multiple quantitative trait loci (QTL) that contribute to trait expression, resulting in phenotypic variation (Todhunter et al. 2005). The mode of inheritance may be quantitative with a major gene affecting the trait jointly with numerous minor genes (Mäki et al. 2004). Hereditary estimates vary from 0.20 to 0.75, depending on radiographic technique, scoring system, breed, differing populations within a breed and different heritability calculation methodologies (Oberbauer, Keller & Famula 2017; Todhunter et al. 2005). Another study found a mean heritability of 0.38 for 15 United Kingdom breeds with only a small degree of heterogeneity among breeds (Lewis et al. 2013). One study in Labrador retrievers had a heritability value of 0.53 (paternal half siblings) (Ohlerth et al. 1998) whilst in other studies, values of 0.35 (Woolliams et al. 2011), 0.26 (Mäki et al. 2002) and 0.59 (Oberbauer et al. 2017) were found. The latter two studies had heritability values of 0.38 and 0.57 for Rottweilers.

Elbow dysplasia is an all-encompassing term comprehensible to the lay public referring to fragmented medial coronoid process (FMCP), osteochondrosis (OC) and osteochondritis dissecans (OCD), ununited anconeal process (UAP) and elbow incongruity (Kirberger & Fourie 1998). These conditions may occur on their own or in combination with each other (Kirberger & Fourie 1998; Meyer-Lindenberg, Fehr & Nolte 2006). Clinical signs are seen from 4 months of age onwards followed by the development of arthrosis, which may be crippling or subclinical. The pathophysiology, diagnosis and control were reviewed prior to introducing an elbow grading system in South Africa to make local veterinarians more aware of the condition (Kirberger & Fourie 1998).

Official ED certification started in South Africa in 1999 and is usually performed in conjunction with HD grading and according to the International Elbow Working Group scheme (IEWG) evaluation criteria (Kirberger 2016). Only a single flexed mediolateral view of each elbow is evaluated. In 2007, the author published an article on the findings of the elbow scheme in South Africa from inception to the end of 2006 (Kirberger & Stander 2007). This study showed that Rottweilers had the highest ED prevalence of 55% with the Labrador retriever prevalence ranked 12th with 21% of dogs graded as dysplastic. The respective figures for the above values in the United States at that time ranked Rottweilers second (prevalence 41%) and Labrador retrievers ranked 24th (prevalence 12%), nearly half of the prevalence of HD and ED in these two breeds in South Africa.

The IEWG elbow certification process evaluates for signs of elbow arthrosis, the consequence of the various components of ED. Osteophyte formation at very specific locations within the elbow joints are evaluated for size and are graded from 1 to 3, with 0 being a normal joint. In more recent years, if a primary cause is visible, the cause is stated and the grading is automatically 3. Suspicion of a primary lesion is graded 2 (Ohlerth et al. 2016). The minimum requirement for IEWG grading is a 45° flexed, well collimated mediolateral (ML) view of both elbows, and this has been the only view required in South Africa since inception of the scheme. With the development of digital imaging systems, a single 100° – 120° ML flexed view may be more advantageous to define medial coronoid process pathology, as digital manipulation of the image will still allow visibility of osteophytes on the anconeal process, which is superimposed on the medial epicondyle on this view (Ondreka & Tellhelm 2017). Various countries insist on additional views to improve interpretation accuracy for osteophyte and primary lesion detection, and this accuracy increases with the number of views made. It is thus important to realise that, from a grading perspective, OCD and subtle FMCP changes are usually not visible on a single flexed ML view, and if a diagnosis needs to be made for clinical evaluation of the pathology, multiple views are required (Kirberger 2016).

In South Africa, these ED evaluations have been done by individual specialist veterinary radiologists as part of the SAVA/KUSA HD/ED scheme. The ED scheme has been in operation for nearly 18 years, and it was decided to evaluate the elbow results in Rottweilers and Labrador retrievers since 2007, to follow on the previous South African elbow publication (Kirberger & Stander 2007) to determine whether progress has been made over the last 9 years in reducing the incidence of ED in the current group of dogs being examined.

Elbow dysplasia is one of the more common heritable orthopaedic conditions, with the Rottweiler being the only breed in which the existence of a major gene has been suggested (Mäki et al. 2004). A recent study in 60 breeds showed ED heritability to range from 0.01 (Cavalier King Charles spaniel) to 0.90 (Welsh springer spaniel) (Oberbauer et al. 2017). Males may have a higher heritability than females (Kirberger & Stander 1998; Lang et al. 1998; Oberbauer et al. 2017). More recently, the heritability of elbow scores in Rottweilers was found to be 0.14 (Lewis et al. 2013), 0.37 (Mäki et al. 2002) and 0.68 (Oberbauer et al. 2017) and in Labrador retrievers 0.10 (Mäki et al. 2002; Oberbauer et al. 2017) and 0.19 (Woolliams et al. 2011). The various components of ED may be inherited independently from each other (Janutta et al. 2006; Stock et al. 2011). It has been shown that breeding affected dogs with each other will result in a higher incidence of offspring ED when compared with normal dogs being bred with each other (Keller et al. 2011). It is also important to be aware of the fact that certain breeds are predisposed to ED and its various components, for example, German shepherd dogs to UAP and Rottweilers and Labrador retrievers to FMCP (Kirberger 2016). Environmental factors also play a role in the development of ED and the subsequent arthrosis. These factors include overfeeding (i.e. high body weight), high fat intake, excessive calcium and short bursts of exercise up to the age of 24 months (Sallander, Hedhammar & Trogen 2006).

Selecting for HD in Labrador retrievers has been shown to have a positive influence in reducing ED with a genetic correlation between the two traits of 0.41 ± standard error 0.09 (Lewis et al. 2011; Woolliams et al. 2011). In German shepherd dogs, a moderately positive correlation of 0.3 was found between HD and ED (Stock et al. 2011). The presence of HD and ED is thus genetically related and selection may be mutually beneficial.

The objectives of this study were to determine whether there were any changes over time in the HD and ED prevalence and radiological score over the 9-year study period and if elbow scores had improved since the previous study that was completed in October 2006. We hypothesised that Rottweilers with a prescribed minimal allowable HD grade for breeding would show greater improvement in phenotypic hip status versus the Labrador retrievers, which have no HD or ED breeding restrictions and rely solely on the owner to make the choice of which dogs to breed with, some of which may not have been certified. There are no prescribed ED breeding requirements for dogs in South Africa, but it is compulsory for Rottweiler owners to have their dogs certified for HD and ED prior to breeding. The author hypothesised that there may be a slight reduction in ED incidence from 2007 to 2015, perhaps more so in Rottweilers, owing to self-selection of breeding dogs, as HD and ED gradings are recorded on the KUSA registration certificates.

## Materials and methods

This study examined the Rottweiler and Labrador retriever records of most HD and ED certification radiographs evaluated by official KUSA scrutineers from 01 January 2007 to 31 December 2015. Data were retrieved from the KUSA database. Missing earlier KUSA data were collected primarily from the author’s personal records and those of Faculty of Veterinary Science, University of Pretoria, where many certifications were also done by official scrutineers whilst employed by the University. Only dogs bred in South Africa and evaluated by South African scrutineers were evaluated.

The breed, date of birth, date of certification, age in months, sex, the side affected, HD/ED grading as well as scrutineer name were recorded. Age data were skewed and hence median and interquartile ranges were reported. No attempt was made to determine the primary cause of ED. To see if a reduction in age at certification occurred, 2 year groups (2007–2011 and 2012–2015) were compared with each other. Using HD categorical data, the incidence of HD was determined for each breed and if there were differences between males and females and left and right joints. Categorical HD data were recorded as normal (Grades A1–B2) or dysplastic hips (Grades C–E) for each individual joint. The marginal hip grade of B2 was taken as normal for this comparison. Incidence for ED was taken as normal (Grade 0) or dysplastic elbows (Grades 1–3). An overall normal or dysplastic value was then given to each dog by taking the highest individual grade or score of the left and right joints to determine if the dog was dysplastic or not (e.g. a dog with a Grade 0 left elbow and a Grade 1 right elbow was thus graded as dysplastic). In addition for HD evaluation, the grading was given a numerical score from 0 for A1 to 9 for E2 giving a maximum worst score for each dog of 18 to work with, resulting in numerical rather than categorical data as has similarly been described for OFA data (Keller et al. 2011). The HD and ED numerical grading mean and standard deviations (SDs) were calculated to one decimal point for categorical data and two decimal points for numerical data per breed, sex and for the left and right joints individually and summed to give a total score for the combined joints for the 9-year period. In addition, the total numerical scores were determined for each year individually to see if an improvement had occurred in grading scores for the 2 year groups (2007–2011 and 2012–2015). Incidence data, including sub-grades for dysplastic hip dogs (C–E) were then compared to similar data obtained directly from the OFA offices for each individual year and for the whole period to compare South African findings with those of an extensive and long running database from another country. In addition, the number of registrations of each breed that could have been registered, and been old enough to be certified for each year, were obtained from the KUSA data to get an idea of what percentage of dogs were actually graded for each breed. A possible correlation between the presence of HD and ED in the same dog was also sought for each breed.

Possible scrutineer grading differences were sought between the scrutineer with the most evaluations, scrutineer with the second most evaluations and the remainder of the scrutineers combined as a group owing to low numbers.

Statistical analysis was undertaken using the Stata program version 14 (StataCorp LLC, Lakeway drive, College Station. Texas, USA). Data were mainly descriptive. Differences in the incidence of HD between all dogs, as well as males and females for each breed were determined using the one-sided Fischer exact test. Differences in the HD and ED numerical scores of all dogs as well as males and females were determined using the Mann–Whitney–Wilcoxon test. Reduction in age at certification and improvement in numerical total scores over time by comparing years 2007–2011 as a group with years 2012–2015 were evaluated using the Mann–Whitney–Wilcoxon test. The Pearson’s chi-square test of association was used to determine if there was a statistically significant association between the presence of both HD and ED in individual dogs. Comparison of scrutineers was done by using the Dunn test which incorporated a Kruskal–Wallis and post-hoc pairwise comparisons with a Bonferroni correction for multiple testing. Significance was taken at *p* < 0.05.

## Results

**FIGURE 1:**
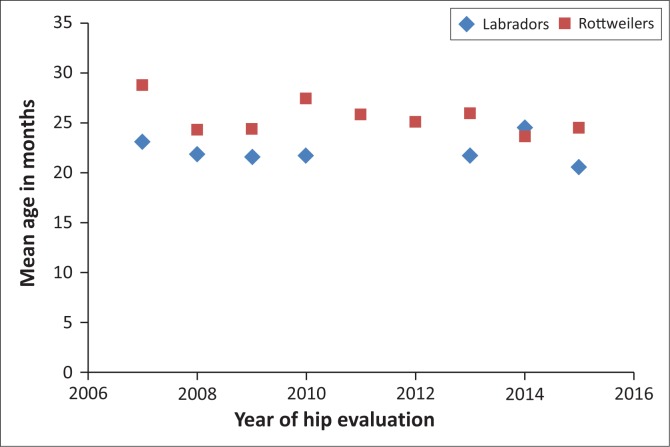
Mean age in months at hip examination in Rottweilers and Labrador retrievers per year from 2007 to 2015.

Data from 2057 dogs were evaluated. For both breeds, there were close to twice as many females as males. The median certification age for Rottweilers was 22 months and for Labrador retrievers it was 18 months, with only minor differences in age between males and females. When comparing mean age for years 2007–2011 as a group with years 2012–2015, there was a statistically significant reduction in the age of certification for Rottweilers (*p* = 0.002) but not for Labrador retrievers (*p* = 0.452) (Figure 1).

The prevalence of HD in the two breeds of the study population for left and right hips separately, combined, as well as for males and females are given in Table 1a. Labrador retrievers had a 9% higher HD prevalence than Rottweilers. There was no significant difference between males and females (Rottweilers *p* = 0.393; Labrador retrievers *p* = 0.299) although females tended to have a slightly higher overall prevalence than males. The numerical HD scores are given in Table 1b. Rottweilers had a mean total hip score of 3.94 and Labradors 5.02 with in both breeds left hips having a slightly higher score than right hips. There was no significant difference between males and females (Rottweilers *p* = 0.085; Labrador retrievers *p* = 0.515) although females tended to have a slightly higher scores than males.

**TABLE 1a:** Prevalence of hip dysplasia in Rottweilers and Labrador retrievers and in males versus females and primary versus secondary versus remaining scrutineers for left, right and both hips using categorical data (Grades A and B, normal; Grades C–E dysplastic).

Discriminator	Rottweilers				Labradors			
	*N*	% dysplastic			*N*	% dysplastic		
		Left hip	Right hip	Dog		Left hip	Right hip	Dog
Total	1141	16.7	17.4	22.3	892	26.6	24.2	31.1
Males	410	18.5	15.4	22.3	286	25.9	23.4	31.9
Females	731	15.9	18.5	23.1	606	26.9	24.6	32.7
Scrutineer 1	715	16.4	18.0	22.7	585	24.6	23.8	29.2
Scrutineers 2	251	13.6	12.8	16.7	78	26.9	23.1	32.1
Scrutineers 3–8	173	22.5	22.1	28.9	167	33.5	25.2	37.1

**TABLE 1b:** Mean hip dysplasia grading score in Rottweilers and Labrador retrievers and in males versus females and primary versus secondary versus remaining scrutineers for both hips using numerical data (0–9 per hip).

Discriminator	Rottweiler		Labrador	
	*N*	Mean score	*N*	Mean score
Total	1141	3.94	892	5.02
Males	410	3.79	286	5.00
Females	731	4.02	606	5.02
Scrutineer 1	715	3.92	585	4.85
Scrutineer 2	251	3.38	78	4.68
Scrutineers 3–8	173	4.86	167	5.65

The prevalence of ED in the two breeds of the study population for left and right elbows separately, combined, as well as for males and females are given in Table 2a. Rottweilers had a 19% higher ED prevalence than Labrador retrievers. Rottweiler males had a significantly higher ED prevalence (8%) than females (*p* = 0.005) whilst in Labrador retrievers there was no significant difference (*p* = 0.433). The numerical ED scores are given in Table 2b. Rottweilers had a mean total elbow score of 1.04 compared with 0.51 in Labrador retrievers. Rottweiler males had a significantly higher ED score than females (*p* = 0.005), but in Labrador retrievers this was not the case (*p* = 0.603) although males did have higher scores than females.

**TABLE 2a:** Prevalence of elbow dysplasia in Rottweilers and Labrador retrievers as well as in males versus females and primary versus secondary versus remaining scrutineers for left, right and both elbows (Grade 0, normal; Grades 1–3, dysplastic).

Discriminator	Rottweilers				Labradors			
	*N*	% dysplastic			*N*	% dysplastic		
		Left elbow	Right elbow	Dog		Left elbow	Right elbow	Dog
Total	1041	33.1	32.8	39.1	877	15.4	15.4	19.8
Males	373	37.5	37.4	50.1	285	16.5	16.8	23.1
Females	668	30.5	30.2	41.9	592	14.9	14.7	22.3
Scrutineer 1	632	35.8	34.5	41.5	583	14.6	14.2	18.7
Scrutineers 2	243	21.8	22.2	28.0	77	08.5	06.5	07.80
Scrutineers 3–8	164	38.4	37.8	45.7	167	24.6	24.6	31.1

**TABLE 2b:** Elbow dysplasia grading score in Rottweilers and Labrador retrievers as well as in males versus females and primary versus secondary versus remaining scrutineers for both elbows (0–3 per elbow).

Discriminator	Rottweiler		Labrador	
	*N*	Mean score	*N*	Mean score
Total	1041	1.04	877	0.51
Males	373	1.26	285	0.57
Females	668	0.92	592	0.48
Scrutineer 1	632	1.15	583	0.47
Scrutineer 2	243	0.69	77	0.26
Scrutineers 3–8	164	1.11	167	0.83

**FIGURE 2:**
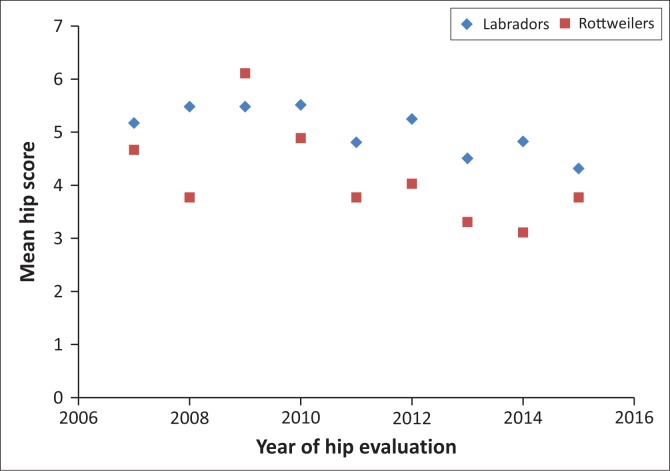
Mean total numerical score (0–18) of hip dysplasia in Rottweilers and Labrador retrievers per year from 2007 to 2015.

**FIGURE 3:**
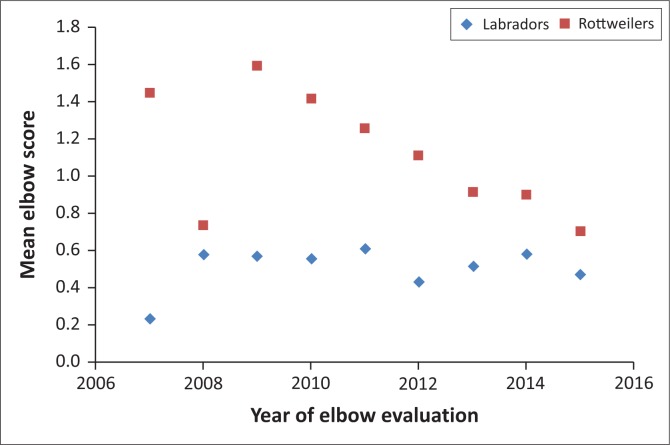
Mean total numerical score (0–6) of elbow dysplasia in Rottweilers and Labrador retrievers per year from 2007 to 2015.

The HD and ED numerical scores for individual years showed a reduction for Labrador retriever HD and Rottweiler ED scores over time (Figures 2 and 3) but not for the other parameters. In comparing findings for the two age groups (2007–2011 and 2012–2015), significant improvement only occurred in the later years for Rottweiler HD and ED numerical scores (*p* = 0.000 and *p* = 0.000, respectively), but Labrador retrievers only showed a significant improvement in the HD numerical score (*p* = 0.013) with ED showing no improvement (*p* = 0.769).

A highly significant association was found in the Rottweilers (*p* = 0.001) that dogs with HD could also have ED, with 137/1139 dogs having both HD and ED and 511 dogs having no HD or ED. Thus, there was a greater chance of Rottweilers with either HD or ED having both conditions than only having HD or ED. Interestingly, there was no association found between the presence of HD and ED in the Labrador retrievers (*p* = 0.259).

The prevalence of HD being absent or present, including dysplastic subcategories of KUSA dogs in South Africa compared to OFA dogs in the United States, are given in Table 3. The incidence of Rottweiler dysplastic hips was initially much higher in KUSA dogs than OFA dogs but over time the KUSA values approached the OFA values. The mean number of dysplastic Rottweiler hips over the 9 years was 6% higher in the KUSA dogs. However, the mean number of dysplastic Labrador retriever hips over the 9 years was 21% higher in the KUSA dogs, with no obvious improvement in either country over the 9 years.

**TABLE 3:** Grade incidence of dogs with hip dysplasia (Grades C–E) in South Africa with data compared to that of the Orthopaedic Foundation of America from 2007 to 2015.

Breed	Year	*N*		% Normal: Grades A–B		% Dysplastic					
						% Grade C		% Grade D		% Grade E	
		SA	USA	SA	USA	SA	USA	SA	USA	SA	USA
Rottweiler	All	1140	9735	78†	82	10	11	8	6	4	1
	2007	94	1260	69	81	15	12	10	6	6	1
	2008	50	1254	86	83	8	9	4	7	2	1
	2009	49	1059	61	84	18	11	14	4	6	1
	2010	125	1036	71	82	17	11	7	7	5	0
	2011	158	1074	78	82	11	12	6	6	4	1
	2012	149	1066	75	85	12	9	10	5	3	1
	2013	152	952	81	80	8	14	10	6	1	1
	2014	182	1026	85	82	6	12	5	5	3	0
	2015	181	1008	80	82	4	12	11	6	5	1
Labrador	All	891	60244	69	91	9	12	6	11	2	8
	2007	84	8427	71	89	8	8	6	3	14	1
	2008	61	7857	72	89	5	7	13	3	10	0
	2009	99	6886	70	90	8	6	14	3	8	1
	2010	111	6404	68	91	10	6	12	3	11	0
	2011	114	5741	69	91	14	6	7	2	10	1
	2012	90	5936	62	92	16	6	19	2	3	0
	2013	146	5999	64	92	21	6	9	2	5	0
	2014	104	6196	71	92	11	6	8	2	11	0
	2015	82	6778	77	91	7	6	11	2	5	0

† , Percentages may not always add up to 100% because of rounding of values.

The prevalence of ED as normal and dysplastic, including dysplastic subcategories of KUSA dogs compared to OFA dogs, is given in Table 4. The incidence of Rottweiler dysplastic elbows was initially much higher in KUSA dogs than OFA dogs but over time the KUSA dogs approached the OFA values. The mean number of dysplastic Rottweilers over the 9 years was 4% higher in the KUSA dogs. However, the mean number of dysplastic Labrador retrievers over the 9 years was 22% higher in the KUSA dogs, with no obvious improvement in either country over the 9 years. Note that six times more Labrador retrievers were evaluated for HD and ED than Rottweilers in the United States compared to Labrador retrievers being 8/10ths of Rottweilers examined in South Africa.

**TABLE 4:** Grade incidence of dogs affected by elbow dysplasia (Grades 1–3) in South Africa with data compared to that of the Orthopaedic Foundation of America from 2007 to 2015.

Breed	Year	*N*		% Normal: Grade 0		% Dysplastic					
						% Grade 1		% Grade 2		% Grade 3	
		SA	USA	SA	USA	SA	USA	SA	USA	SA	USA
Rottweiler	All	1041	7278	61†	65	17	28	20	7	4	1
	2007	45	799	49	61	18	30	29	7	4	1
	2008	37	861	73	61	11	30	16	8	0	1
	2009	39	761	46	63	21	30	28	7	5	1
	2010	107	775	48	68	21	24	27	7	4	1
	2011	153	811	56	65	16	28	22	6	6	2
	2012	137	849	58	65	18	29	20	5	4	1
	2013	160	766	64	67	17	26	16	7	3	1
	2014	184	834	63	66	20	28	13	6	4	1
	2015	179	822	73	65	14	26	10	7	3	1
Labrador	All	877	41344	80	91	9	6	7	2	2	1
	2007	79	4665	90	91	6	7	3	2	1	1
	2008	63	4665	78	90	13	8	5	2	5	1
	2009	89	4316	78	90	10	8	8	2	4	1
	2010	112	4295	77	91	13	6	6	2	4	1
	2011	111	4094	78	91	5	7	12	2	5	1
	2012	87	4399	80	91	8	7	8	2	3	1
	2013	148	4563	80	91	7	7	8	2	4	1
	2014	107	4825	79	93	8	5	7	2	6	0
	2015	81	5532	85	91	6	6	2	2	6	1

† , Percentages may not always add up to 100% because of rounding of values.

The number of dogs certified for HD as a percentage of registered dogs was evaluated for years 2008–2013 and compared between KUSA data and that of the OFA. For Rottweilers, the mean value was 29% in the United States compared to 16% in South African dogs. The figures for Labrador retrievers were 3% and 6%, respectively.

The number of certifications done by each individual scrutineer as well as three groupings is given in Tables 1 and 2. Differences between total number of dogs evaluated by the scrutineers and total number of certifications were because of the name of the scrutineer not being available for some dogs, particularly of earlier Labrador retriever data. Scrutineer 2 had significantly lower gradings for HD evaluations than scrutineer 1 (*p* = 0.0007) and the remaining scrutineer group (*p* < 0.0001). There was no significant difference between scrutineer 1 and the remaining scrutineers (*p* = 0.1359). Similarly for ED evaluations, Scrutineer 2 had significantly lower gradings for ED evaluations than scrutineer 1 (*p* = 0.0133) and the remaining scrutineer group (*p* = 0.0001). There was no significant difference between scrutineer 1 and the remaining scrutineers (*p* = 0.5012).

## Discussion

This study examined a very select group of Rottweilers and Labrador retrievers that were presented for HD and ED certification and as such results cannot necessarily be extrapolated to all dogs in these two breeds. An additional potential bias is that owners of dogs presented to their local veterinarian for radiography may not always have submitted the radiographs for certification if the dog is obviously affected by HD or ED. However, most similar studies suffer from the same constraints and data are thus analysed to provide local veterinarians and breeders with information that can be useful in the setting of breeding guidelines.

In our study population, South African Rottweilers had a lower HD prevalence than Labrador retrievers as well as lower numerical scores. This finding was contrary to the prevalence of HD in the OFA data, where Labrador retrievers had about a 10% lower prevalence than the Rottweiler.

This can be ascribed to the much higher prevalence of HD in South African Labrador retrievers, which exceeds that of its US counterparts by about 20%. However, Labrador retrievers had a lower prevalence of ED than Rottweilers but again this was about 10% higher than seen in the United States.

The South African data were compared to that of the OFA to show our breeders where they stand in relation to a large American data base. Cognisance must be taken of differences between the two grading systems. In South Africa, Labrador retrievers are graded from 12 months onwards (median age in this study was 18 months) and Rottweilers from 18 months onwards (median age in this study was 22 months), whereas the OFA certifies dogs for HD and ED from 24 months of age. Thus, the prevalence of HD and ED in the South African populations could have been underestimated. Despite the above differences, it is obvious from Tables 3 and 4 that the incidence of HD and ED in KUSA Labrador retrievers far exceeds that found in OFA dogs. Unfortunately, there is no comparative study available on the prevalence of HD in the South African population prior to 2007. For Labrador retrievers, despite the small numbers and low selection pressure applied, they did show a minor but significant decrease in the HD numerical scores between the two age groups (2007–2011 and 2012–2015). However, there was little change in the number of dysplastic dogs in these two periods. This reflects poorly on the Labrador retriever breeding practices, breed society management and even KUSA, and appears to be because of negligible selection practices for HD and ED. The current study only looked at Labrador retrievers as an example of a breed in South Africa with no HD or ED breeding restrictions and as such there are likely to be many more breeds in South Africa that are in exactly the same situation. This emphasises the need for most breeds to implement or enforce HD and ED certification and set minimum standards for which grades can be used for breeding. Contrary to this, the KUSA Rottweilers started off with about a 10% higher HD incidence than their OFA counterparts but towards the end of the study the incidence was very similar to that in the United States. This is also reflected in the significant decrease in the HD (and ED) numerical scores between the two age groups (2007–2011 and 2012–2015). This can be attributed to their requirement that all dogs must be certified for HD and ED prior to breeding and that only dogs with an A or B grading can be used for breeding and if a C grading is used, it must be bred to an A or B grade of the opposite sex. The same trend could be seen for Rottweiler ED scores despite having no specific minimum ED grading breeding requirement. This can be attributed to the genetic correlation between HD and ED as shown in Labrador retrievers with the selection of one trait having a beneficial effect on the other trait (Lewis et al. 2011; Woolliams et al. 2011). Interestingly, this was not found in the Labrador retrievers in our study, possibly because of lower numbers. However, our Rottweilers did have a positive correlation between the presence of HD and ED. Interestingly, the only difference between males and females in the study was that Rottweiler males had significantly higher ED scores than females. In our previous ED study, males in general had a significantly higher ED score than females. This could be attributed to their faster growth rate and higher body weights (Guthrie 1989; Guthrie & Pidduck 1990).

In comparing the prevalence of ED in the current study to that of the previous South African publication for the time period 1999–2006 (Kirberger & Stander 2007), Rottweilers at that stage had a mean ED prevalence of 54.7%, which over the current period decreased to 39.1% and had decreased to 27% by 2015. This is a great improvement and can probably be ascribed to setting breeding criteria as mentioned earlier. In contrast, 20.6% of Labrador retrievers assessed during the 1999–2006 period had ED. It continued to affect 19.8% of Labradors in the current study period although it did end at 15% in 2015. From these data, it appears likely that over the last 16 years, the Labrador retriever population in South Africa has seen minimal improvement in the prevalence of ED.

A limiting factor in decreasing the incidence of ED is that the technique of a single flexed radiographic view, especially if maximally flexed, will underestimate the incidence of ED, particularly FMCP. Some dogs may have the primary disease condition without any sign of arthrosis and therefore some of these will be missed if additional views, including a 100° – 120° ML flexed view, or more advanced imaging systems are not utilised. It has been found that computed tomography (CT) detects many more ED changes than radiographs because of it being a cross-sectional imaging technique without any superimposition of osseous structures (Kunst et al. 2014; Lappalainen et al. 2009). Up to 62% of dogs graded as 0 based on radiographs were still found to have dysplastic changes in the elbows using CT. The latter figures, however, also depend on the experience of scrutineers and the number of views. Experienced versus inexperienced scrutineers had a > 92% sensitivity to detect medial coronoid disease radiographically using three views as compared to CT to detect pathology (Rau et al. 2011). Grading dogs as normal during radiographic screening when they are positive on CT obviously has serious implications for the radiographic certification method in that affected dogs may be graded as ED-free, thus hampering elimination of ED genes. Unfortunately, the use of CT is not a cost-effective and practical method of grading dogs for ED certification, and radiographs will continue to be used, but will at least exclude the more severe cases from the breeding population.

Scrutineer 2 appeared to be significantly more lenient in grading dogs than the remainder of the scrutineers. In order to prove this, one would have to have the same radiographs interpreted by all the scrutineers, which was not the objective of the current study. It may well have been that scrutineer 2 had better dogs to grade. However, the subjective nature of interpreting the radiographs for HD is well documented (Verhoeven et al. 2007, 2009). Using the FCI scheme and European observers’ inter-observer agreement distinguishing between dysplastic and non-dysplastic dogs was 72%, which increased to 76% for experienced scrutineers. For providing a final score (A, B, C, D or E) inter-observer agreement was only 42%. In addition, rejection of radiographs according to the technical quality and accuracy of positioning also differed between these scrutineers. The OFA, however, claims agreement between normal, borderline and dysplastic hips for their differing scrutineers to be 93% – 95% (Verhoeven et al. 2012). One reason for the difference between FCI and OFA data could be that the latter scrutineers represented only one country and all were radiologists, which is similar to the situation in South Africa. In South Africa, which follows the FCI grading system, results can be appealed and the radiographs evaluated by a different scrutineer. The appeal results may be the same, better or worse that the original result, with the best result being accepted as the final grading by the KUSA. It is thus theoretically possible that a C1 could be regraded to B1, which may allow a dog to breed in those breeds that have breeding restrictions, but it is highly unlikely that a D2 will be regraded to a B1 to enable breeding. It is the author’s opinion that it is a pointless exercise to appeal dogs with a D or E grading as they definitely are dysplastic, and even improving a grade to a C still grades the dog as dysplastic and it should not be used for breeding.

Dogs suffering from HD and ED, especially in the more advanced stages, can suffer severe pain and mobility impairment, disabling the dog and affecting its welfare (Mäki et al. 2004; Stock et al. 2011). Besides the suffering caused, treatment is also very expensive, requiring long-term medical treatment and often extensive and very expensive surgery. Implementing breeding restrictions will help to reduce the prevalence of HD and ED as can be seen in this study and many others. Progress is unfortunately slow but we owe it to our dogs to minimise their chances of getting a potentially debilitating orthopaedic disease. What should veterinarians, breed societies and dog breeders in South Africa do to improve the HD and ED health status of their dogs? When considering which dogs to breed with each other, the grading of an individual dog should be combined with that of vertical (depth-of-pedigree, i.e. sire and dam, etc.) grading as well as horizontal (width-of-pedigree, i.e. siblings) grading to improve response to selection (Keller et al. 2011). This can readily be done by any breeder in South Africa. A more sophisticated methodology is by making use of an estimated breeding value (EBV) as provided by some breed societies in overseas countries. The EBV is an estimate of genetic liabilities obtained after removing as far as possible the environmental influences by using an individual’s phenotype and that of its relatives (Lewis, Blott & Woolliams 2010a). A recent HD and ED study of 15 breeds in the United Kingdom showed slow progress in all dogs except the Siberian husky, but including the Rottweiler and Labrador retriever, when using only the phenotypic radiographic score for selection choice. However, the study demonstrated substantial improvement in selection accuracy in all 15 breeds when using an EBV (Lewis et al. 2013). However, an accurate EBV does require a lot of data and this requires the birth of all litters to be officially recorded, and all dogs to undergo standard radiographic examination and certification, which implies having to wait till the dog is at least 1 year old. Besides needing extensive expertise to develop an EBV database in a country, the low percentage of registered dogs in some breeds undergoing certification and the potentially biased population (bad hips not submitted for certification) does not make using EBV a viable option in South Africa at this stage.

The current study was done on two KUSA breeds and the KUSA is the only organisation in South Africa that records the HD and ED certification results, if available, on a registration certificate. Organisations that do not belong to the KUSA such as a variety of Boerboel organisations, field trial dogs and German shepherd dog (GSD) Federation dogs should, as a minimum requirement, follow the KUSA’s example and record HD and ED results on their registration certificates so that owners can make an informed decision as to which dogs to breed with. Dogs at high risk for HD or ED should have a recommendation relating to a minimum grade of HD or ED acceptable for breeding. Currently, only seven breeds (Alaskan malamute, Dobermann, German shepherd dog, Giant schnauzer, Rhodesian ridgeback, Rottweiler and Weimaraner) belonging to the KUSA have such HD requirements. Minimum ED grading should be instituted as a matter of urgency for these breeds as well, including those breeds with a low incidence of ED. There are many other working dogs in South Africa that are at risk, including the various retrievers, Boerboel, German shepherd dog, and others. These breeds should also implement minimum HD and ED requirements, which initially could be quite lax and become more stringent over time, to make some improvement. It can be clearly seen from this study that Rottweilers with a minimum HD grading breed requirement have made more progress over the last 9 years to improve the HD status of the breed when compared to the Labrador retrievers. Interestingly, the Rottweilers also made progress with the incidence of ED despite there being no specific ED grading requirements. Besides the genetic correlation between the incidence of HD and ED and relatively high heritability, the author believes that having HD breeding restrictions and HD and ED radiological certification will result in breeders learning more about these conditions, ensuring better breeding practices.

In addition, breed societies as well as the FCI should also reconsider their interpretation of individual hip scores and grading on the worst score. Recent research from the British Kennel Club based on Labrador retrievers, still to be confirmed in other countries and breeds, showed that a dog’s left and right hip scores have nearly identical genetic parameters and that utilising the worst score only adds bias because of environmental impact (Lewis, Woolliams & Blott 2010b). The total score or average of two scores should rather be used. This is easier in cases where a numerical score is given to each hip, as in the BVA scheme, but as an example, in South Africa, where scores range from A1 to E2 and the hips differ by two sub-grades, the mean grade could be used to decide on breeding. In South Africa, a Rottweiler bitch, for example, can only breed a worst hip score of C2 with a worst grading of B2 male. However, if she had a left D1and right B1 grading, the mean would be C1, thus allowing the bitch to be bred to a B2 male. In addition, in a breed such as the Rottweiler that has made progress in improving the phenotypic HD status over the last few years, the minimum grade could be raised from a C2 to a C1 for breeding to ensure that improvement will continue.

Improving the accuracy of grading by scrutineers to improve phenotypic selection should also be considered. Possibilities include having at least two scrutineers evaluating each case and reaching a consensus opinion; for ED having at least two views of each elbow (100° – 120° ML flexed and pronated CrCd views) and for HD to consider introducing an additional simple HD distraction view. Unfortunately, all of these will have cost implications for breeders.

Screening dogs for HD is not only done to determine a phenotype for future breeding but also to predict HD-related clinical signs for the individual dog. The latter has cost implications, as it has been shown that worsening dysplasia will lead to a significantly higher incidence of veterinary care and mortality (Malm et al. 2010). Non-breeding predisposed dogs should thus also be encouraged to have HD and ED certifications performed, not only to assess the status of their joints from a clinical point of view but to also contribute to the breed genetic data in order to determine an EBV for their relatives.

## Limitations of the study

The study was limited to 2057 dogs of two breeds and increasing the number of dogs or the number of years could have resulted in improved statistical efficacy. The low percentage of available dogs evaluated, particularly for the Labrador retriever (6%), may also have skewed the data, and the findings in our study population may thus not be representative for the breed population as a whole. Having more than one scrutineer evaluate the radiographs and getting a consensus opinion on the HD or ED grading would no doubt result in improved accuracy of grading results. The author is a radiologist and not a geneticist and hence data evaluation concentrated on the incidence of HD and ED for the time period. Genetic analysis of the pedigrees of the dogs included in this study would have been very beneficial, but this has to be left to the KUSA or individual breed societies to instigate to eventually get an EBV for breeding dogs in South Africa, providing the majority of breeding dogs are radiographed and certified.

## Conclusion

This study has shown that in Rottweilers, as an example of a breed applying selective breeding based on HD results, progress can be made in reducing the incidence of HD and ED. On the opposite side of the spectrum, in Labrador retrievers as an example of not having any breeding restrictions, only minimal HD progress was made in alleviating the incidence of these two often crippling orthopaedic conditions. In order to reduce the effect of HD and ED in the various South African dog breeds, particularly in working dogs, stringent breeding guidelines must be put into place. Breeds predisposed to HD or ED that have no breeding guidelines should have these instituted as soon as possible. Breeds that already have guidelines in place can consider making the HD breeding guidelines stricter by a sub-grade and introduce ED breeding restrictions. In addition, it can also be considered to at least impose stricter ED breeding restrictions for males, which in the current study had a higher incidence of HD and ED when compared to females and produce more offspring than the females. The next step is then to employ suitably qualified personnel to determine an EBV for the breeds at risk to enable better future genetic selection to reduce the incidence of HD and ED.
